# The London Lancet, American Reprint

**Published:** 1873-03

**Authors:** 


*The London Lancet, American Reprint.*

Through the courtesy of W. C. Herald, publisher, No. 52 John street, New York, we are supplied with the monthly issues of this sterling old journal.

